# Monographie des Degenerations Skirrheuses de l'Estomac, &c.

**Published:** 1817-12

**Authors:** 


*Monographie des Degenerations Skirrheuses de l'Estomac, &c.* Par FREDERIC CHARDEL, D. M. &c.

( Continued from page 218. )

HAVING reviewed in our 21st Number, Dr. Chardel's valuable collection of anatomical facts relative to this subject, we now proceed to investigate

*The* CAUSES *of Scirrhous Affections of the Stomach.* These, although far from being always evident, may generally be referred to Mechanical Irritation; Chemical or Animal Irritation, and a peculiar State of the Lymphatic System.

1. *Mechanical Irritation.* Under this section are ranged occupations, requiring pressure on the epigastrium; enlargement of adjacent organs mechanically incommoding the stomach; and the effects of external violence. From a cautious retrospect of facts, mechanical pressure seems not to exert the influence, which has been supposed, in the production of gastric scirrhus: for the disease attacks indiscriminately every description of artizans; and, rarely observed in the anterior part of the stomach which is most exposed to pressure, in general affects portions of the organ evidently best sheltered from it—the cardia, curvatures, and, especially, the pylorus. Should the stomach, however, suffer from this cause, it probably occurs rather through the medium of the gastric disorder induced by, than as a direct consequence of, the mechanical pressure. In the latter case, the abdominal parietes, interposed between the external agents and the stomach, must necessarily have sustained the brunt of their influence ; and will hence share the local injury, from which they have served, in some degree, to protect the digestive organ. Of this we have here an example : A hatter had a tumour, produced by constant pressure, in the epigastric region. A gangrenous slough, separating from its centre, gave issue to a putrid matter mixed with aliment. On dissection, the anterior part of the stomach, become scirrhous, adhered to the abdominal paries ; and had degenerated into a cancerous sore. Of all the neighbouring organs, the liver, when enlarged, is most likely to encroach upon the stomach. Dr. Chardel has seen a large hepatic tumour resting on, and compressing, a scirrhous pylorus. An analogous instance is recorded by Morgagni. Yet enlargement of the liver is more frequently an effect, than cause, of gastric scirrhus.

2. *Chemical Irritation.* Agents of this class are either corrosive, and disorganize the parts with which they come in contact; or merely acrid, and produce irritation, inflammation, and tumefaction. The latter only come under consideration here. Mercury, injudiciously employed in the cure of gonorrhœa, has been known to induce ulceration of the stomach with contracted pylorus. Chronic inflammation, thickening and fungus, of the membranes may be also the consequences of unsubdued irritative action. Persons, addicted to the abuse of spirituous liquors, and even they who drink such liquors early in a morning, are not more subject than others, to scirrhus of the stomach. A contrary opinion has frequently been advanced ; but it seems not to rest on the basis of sound observation.

3. *Animal Irritation.* By animal irritants are meant the various morbific humours generated or developed in the human body. Dr. Chardel here combats, with considerable ability, the doctrine of purulent metastases. Like many eminent pathologists of the present day, he refers the production of hectic fever rather to the influence of irritative action than to that of re-absorbed pus; and supports his position by arguments obvious to every one who has thought profoundly on the subject. Some observations on the nature and mode of propagation of the different species of virus follow; and the case of the author's mother, who, after repeated operations for its removal, fell a victim to mammary cancer, is somewhat interestingly, although perhaps unnecessarily, detailed. On the whole, nothing practical or conclusive is advanced in this section; which assumes much more of an hypothetical character than any other portion of the work. Hence it is to us unsatisfactory food; and we quit it with impatience for the more substantial fare that awaits our progress.

4. *A peculiar State of the Lymphatic System.* Scirrhus of the stomach is seldom a purely local disease. The tuberculated condition of the other abdominal viscera, frequently complicated with it, announce, on the contrary, a deep-rooted affection of the lymphatic system, and an analogy of glandular enlargements with the malady in question. Similarity of effect bespeaks identity of cause. Circumstances of every description, which operate in giving an undue predominance to the lymphatic system, serve necessarily to favour the production of glandular enlargement, and, consequently, according to Dr. Chardel's views, of gastric scirrhus also. Of the numerous bodies, annually brought for dissection to *l'Ecole de Médecine*, four in the hundred exhibit scirrhus of the stomach; and this frequency of the affection among the poor, who constitute almost exclusively the subjects of such observation, and who are obviously exposed to the influence of every debilitating cause, confirms the reasonings which have just been advanced, relative to its most common origin.

Remarks *on Scirrhus of the membranes of the Stomach.* Gastric scirrhus generally presents itself under three forms. All of these may exist at the same time: 1. A tubercular state of the omental glands, in the vicinity of the organ, and implicating, more or less, its morbid membranes. 2. Effusion of lymph into the substance of its parietes, offering, on incision, the appearance of coagulated white of egg and forming a tumour of variable magnitude; while the peritoneal coat retains externally its natural polish. 3. Flabby or hardened vegetations, rising from the mucous membrane, and only affecting consecutively, if at all, the peritoneal coat. The affection, in Dr. Chardel's opinion, manifests rather a tubercular,* than a cancerous character. It is not usually attended with the dreadful pains which signalize cancer: nor does the subject of it, to the last, evince extreme sensibility, upon even forcible compression. Yet the disease, when principally affecting the mucous membrane, exhibits greater activity and pain, and more nearly resembles cancer in its progress. Four cases are added in support of this position. The first we shall briefly transcribe. A gardener, aged 37, of robust constitution and sanguineous temperament, regular, and free from any disease to which the fatal complaint could be attributed, felt, in September 1797, severe pains over the whole abdomen, accompanied with nausea and vomiting. Pulsations, spontaneously appeared in the epigastric region. After a fortnight passed at *L'Hotel-Dieu,* the colics subsided; but the pulsation remained. Five months subsequently, the pain reappeared with increased violence; and bilious vomitings ensued. These symptoms were again lulled by sedatives; but the man was shortly obliged to seek assistance at *La Charité,* and went out relieved. Soon afterwards, he was admitted into *L'Hôpital de l'Ouest,* and found benefit from the remedies there employed. He returned, in about two months, to *La Charité;* underwent the treatment for painter's colic; and was dismissed with some relief, in November 1799. To this transient calm succeeded pains more violent than ever; and, in February 1800, he

---

* Tubercles, analogous to those frequently developed in the liver, have occasionally been formed in the encephalon. Dr. Chardel, upon inspecting the body of a female who had died from an affection of the brain, induced by severe injury, and accompanied with paralysis of the left eye, contraction of the corresponding facial muscles, and gradual failure of the right eye, found a tubercle of this description, as large as a pullet's egg, developed on that portion of the dura mater by which the sella turcica is invested. Situated somewhat to the left, this tumour strongly compressed the corresponding optic nerve, which was converted into a sort of ligament; while the right nerve, suffering less from pressure, had not completely lost its functions. The left clinoid processes, and osseous scale, covering the left carotic sinus, were corroded. The interior of the tumour was white, and of a firm and fat-like structure. Small reddish fleshy granulations covered its surface. The brain itself was firm; and its convolutions, as it were, flattened. By pressure on its superior part, a deep-seated fluctuation was perceived; resulting from an effusion of four ounces of serum into the lateral ventricles. There was no congestion of its blood-vessels. Cartilage was deposited between the mucous and the serous membranes of the gall-bladder, so that it could not collapse. Several small concretions, and some colourless bile, perfectly resembling white of egg, were contained in it.

re-entered the hospital. His body was then pale; his countenance of a faded yellow colour; his eyes dim and hollow; his malar bones projecting; and his cheeks deeply sunk in. His mouth exhaled a fetid odour. A blackish and offensive matter was at times ejected by vomiting. Lancinating pains spread through the whole abdomen. Pulsations raised the epigastric region. Obstinate costiveness existed. Sedatives, baths, fomentations, and repeated bleedings, produced no relief. The man's strength was still farther reduced by exhausting diarrhœa: and he died on the 10th of March, in the most dreadful suffering. On dissection, the intestines were found healthy, but shrunk and diminished in volume. The stomach was very large, especially towards its great extremity; and externally unaltered. An ulcer, three inches long, and one and a half broad, with jagged and inverted borders, had corroded the central and posterior part of the organ from within outwards. Intimate adhesions to the inferior surface of the right lobe of the liver had prevented effusion of the aliment into the abdominal cavity. In two of the three remaining cases, which closely resemble the preceding in their anatomical character, distinct epigastric pulsations were observed. Nothing satisfactory is advanced in explanation of this phænomenon.

Ulcers of the stomach are not invariably the consequence of scirrhus. This organ, like all the other parts of the human body, is liable to destruction by the ulcerative process. In proof of this, several cases are adverted to, wherein holes of the stomach, apparently resulting from the action of the absorbent vessels, have been discovered. Hæmatemesis frequently precedes gastric ulceration; and, hence, may sometimes be suspected as its cause. In such cases, the irritation and distention of the blood-vessels of the mucous membrane, induced by habitual plethora, are followed by inflammatory action, which terminates in erosion.

A man, aged 24, of delicate constitution, had been subject, from his sixth year, to vomiting of blood. The attacks were neither frequent nor severe. While serving in the armies of the revolution, he received several blows on the epigastric region, which aggravated the hæmatemesis, and procured his discharge. He felt nothing more of his disease till May 1800, when he was seized with shivering, violent head-ache, and vomiting of blood, more profuse than ever; which soon became intolerably offensive. He sunk, completely exhausted, on the 14th. The body exhaled a putrid odour. On dissection, the stomach was very large, and marked externally by wrinkles, to which corresponded as many dilated blood-vessels. A red violet spot, four inches long and eight lines broad, occupied that part of its surface contiguous to the smaller lobe of the liver and the diaphragm. Another round and inflamed spot, independent of the first, and three inches in diameter, was seen in the interior. The vessels of the mucous membrane were become varicose, and gave issue to the blood on the slightest scratch.*

In the case of a strong man, who had been subject to nasal hæmorrhage in his youth, but had subsequently remained well till near the middle age, the inflammation and ulceration of the stomach appear to have been consequent on sanguineous congestion of that organ. The precursory symptoms, fever, violent head-ache, and epigastric pain were, for a time, removed by medicine. A sense of intolerable prickling and constriction in the œsophagus, followed by profuse gastric hæmorrhage and syncope; discharge of coagulated blood by stool; foul mouth, extreme thirst, nausea, cough, with scanty viscid expectoration; pain in the breast and left hypochondrium; and frequent pulse, stood foremost in the train of phænomena which more immediately preceded death.—Dissection. Sinuses of the brain gorged with blood; slight serous effusion into the ventricles, and into the cavities of the pleura and pericardium. The base of the left lung adherent to the diaphragm. The whole interior of the stomach injected, and of a livid colour. A circular orifice, to the right of the cardia, one inch and a half in diameter, leading to a sort of pouch formed above, by the left lung (the diaphragm being here corroded); externally by the inferior part of the œsophagus and summit of the spleen; and internally by the parietes of the stomach, which, in this place, had acquired the thickness of eight lines. Several livid fungi in the interior of this

* The following case displays an example of fatal hæmatemis, preceded by most of the symptoms of gastric scirrhus. A woman, aged 65, had been for some time affected with vomiting of an acid nature, after food; obstinate costiveness, and emaciation. There was neither tumour nor pain in the epigastrium. Two days after her admission into the Cochin Hospital, she screamed out, complained of a rending pain in the stomach, vomited suddenly a stream of florid blood, and died in about fifteen minutes. On dissection, the œsophagus and stomach, otherwise perfectly sound, were found full of blood. No trace of congestion or rupture of the vessels existed. All the remaining viscera were healthy.

cavity. The diaphragm thickened and scirrhous in the whole circumference of the ulcer. None of the viscera forming the cavity described, altered beyond its limits, with the exception of the stomach.

It may be remarked that, in these cases, as well as in those of spontaneous erosion of the stomach recorded by other authors, vomiting of aliment, and most of the other symptoms of gastric scirrhus, are absolutely wanting.

The lymphatic vessels of the stomach form three principal divisions, which respectively supply the pylorus and the small and great curvatures of the organ: and this mode of distribution is considered as the probable cause by which scirrhus of those parts is so frequently determined. The speculations of Dr. Chardel on the nature and production of the tubercles, with which scirrhus of the stomach is sometimes complicated, present nothing sufficiently attractive to detain us long. The latter appears evidently to be connected with a peculiar state of the lymphatic system; and chemical analysis has shewn that these bodies resemble, in the former respect, albuminous concretions: although, from the fatty character which they often externally present, many authors have described them under the name of steatoma. It has before been observed, that the female sex is less exposed than the male to gastric scirrhus: and this affection very rarely attacks infancy and early youth, at which periods the constitution of the latter approaches more or less nearly to that of woman.

*Of scirrhous Affections of the Stomach in general.*—Gastric scirrhus commonly exhibits three distinct periods; but the disease often proves fatal ere all its phases have been gone through. These periods observe no fixed rule as to their duration, relative or combined. In general, however, the first is the only one in which the disease seems stationary; and hence, it is longer than the two succeeding it, united. The whole, taken together, vary in rapidity of progress, from several months to as many years. Fever rarely manifests itself till towards the close of the malady, when the scirrhus has become ulcerated, or, proceeding with an unusually accelerated stride, it has induced active inflammation.

*The first or incipient stage* is sometimes preceded by a state of general languor and debility. Acidity, flatulence, a sort of constriction which extends from the epigastrium to the hypochondria and back, like a "girdle of pain." From the onset, the pain is fixed, permanent, or of short duration, and more or less acute in the epigastric region. Pressure may perhaps aggravate it, particularly after meals. Exacerbation of the symptoms on the ingestion of food, which obliges the patient gradually to diminish its quantity. Indigestion, accompanied with a sense of weight in the epigastrium, and sometimes vomiting of alimentary matter, which has often an acid taste and smell. Some patients have then recourse to spirituous liquors, in order to assist digestion. At other times, the only symptom is simple nausea after meals; which, gradually increasing, terminates in rejection of the food. Digestion finished, tranquillity is in general restored. Frequently, although the stomach be empty, nausea prevails, and induces an apparently nervous vomiting: and, when the latter does not result, some patients provoke it by the introduction of the finger into the throat, and thus get relief. Diarrhœa, especially when the progress of the disease is rapid; but more commonly torpor and constipation of the bowels. Transient relief generally ensues from the operation of glysters and laxatives. Notwithstanding the gastric disorder, the appetite most frequently remains, or is even increased; and to the craving for food is united a dread of the sufferings consequent on its ingestion. The tongue continues clean and moist, and the taste natural. When foulness appears, it is only an adventitious symptom.

*The second or confirmed stage.* Decided emaciation, often far advanced. An epigastric tumour, varying in extent and in the degree of pain. Reclination on the side opposite to that which the tumour occupies, sometimes painful. Vomiting of food with pain, or by simple rejection, without any change, except that which arises from its having remained for a time in the stomach: it is frequently mixed with a viscous fluid. Patients may pass some days without vomiting when the stomach is not very irritable. The food then accumulates until contraction is induced; and the figure of the distended organ is visible through the abdominal parietes. In this case, it acquires considerable volume, and may offer a sort of fluctuation. Sedatives now frequently produce the happiest effects, and, for a time, arrest the vomiting. The patient, who has been obliged to diminish gradually the bulk and solidity of his food, is often reduced to take only a little milk, broth, or vermicelli. The epigastric pains become almost constant. Obstinate constipation: the fæces hard, scanty, and pitch-like, or resembling meconium. Diarrhœa sometimes alternating with the costiveness. The appetite often unimpaired, and the tongue clean. At times there is an unpleasant taste, although the tongue exhibits no fur.

*The third or ulcerated stage.* Extreme marasmus. The patient is exsanguine, or in a state of œdema. Epigastric pains more severe and permanent. Opium no longer procures sleep. The ejected matter is changed, and often exhales an intolerable smell. At length it assumes the appearance of blackish peas-porridge, or resembles soot mixed with a viscous fluid: sometimes it only presents streaks of this dark colour. The stools undergo similar changes. It may even happen, that these alone exhibit such changes, if the ulcer be situated in the vicinity of the pyloric orifice. All this sometimes takes place without any aggravation of the pain. The breath becomes fœtid, and the mouth very foul: yet the tongue retains its natural condition. Towards the close, diarrhœa frequently succeeds to costiveness; and, not unusually, the vomiting ceases, the stomach having lost its tone. Death takes place without a struggle. Such is the general progress of gastric scirrhus. In some cases, the vomiting, as well as several others of its symptoms, are altogether wanting.

An instance of this kind is shewn in a woman, who was attacked with severe deep-seated pains, striking from the epigastrium to the hypochondria, and aggravated by the ingestion of food, and on the approach of menstruation. From the yellowness of the skin and other bilious symptoms, she was supposed to be suffering from diseased liver. Weakness, irregular fever, erysipelatous eruptions, accompanied with violent itching, œdema, long confined to the left side of the body, and alternate looseness and constipation, formed the other leading phænomena. The patient at length sank, amid dreadful suffering, into marasmus, and died without any recurrence of the diarrhœa. On dissection, the membranes of the stomach were found to possess a cartilaginous induration, and considerable increase of thickness, especially towards its lesser curvature, from the cardia to the pylorus: yet the whole organ, both externally, internally, and in its substance, was quite white, and exhibited no trace of ulcer or inflammation. The pylorus was contracted no farther than by the general thickening of the membranes: the duodenum and other viscera were sound.

Three cases are here introduced, to shew the obscurity in which the diagnosis of organic lesions of the stomach is sometimes involved. We shall transcribe the most interesting.—A cobler, aged 57, who had been, for eight months, troubled with epigastric pulsation, entered *l'hôpital Cochin,* on the 25th of June, 1807. An indolent tumour, extending towards the left, occupied the epigastrium. It exhibited pulsations synchronous with those of the heart, and strongest when the stomach was full: hence, although his appetite was keen, and digestion perfect, he ate sparingly. The countenance was pale, livid, thin, and such as is usually observed in chronic affections of the abdomen. The stools were natural; the pulse strong and regular. The disease was regarded as aortic or cœliac aneurism. Meanwhile, the tumour became painful; the strength failed; copious diarrhœa, and œdema of the lower limbs, ensued. The man died on the 28th of August. On dissection, the thoracic viscera were quite sound. A pint of serum was effused into the abdomen. An enormous scirrhus, ulcerated in the centre, occupied the posterior surface of the pylorus, and half the lesser curvature of the stomach. The pancreas was hard and tumefied; and the liver tuberculated. The intestines displayed their natural volume.

Epigastric pains, progressively increasing in severity, suppressed menstruation, intense jaundice, complicated with ascites, frightful emaciation, loss of strength and appetite, diarrhœa, and slight evening paroxysms of fever, constituted the principal symptoms in a middle-aged female, on whose body the following morbid appearances were unfolded by dissection: An ulcerated scirrhus occupied the anterior surface of the pylorus. The pancreas, also scirrhous, strongly compressed the cysto-hepatic duct. The gall-bladder and biliary vessels were much distended with bile, and the structure of the liver was quite sound. The mesenteric glands were tuberculated; the spleen shrunk; and the intestines much contracted in diameter.

In the case of a mason, aged 50, who, with an epigastric tumour, exhibited all the other external signs of this dreadful malady, and in whom, after death, a large unulcerated scirrhus, occupying the posterior surface of the pylorus, and almost obliterating its orifice, was discovered, the vomiting, at first frequent, after a time totally subsided.

*Do scirrhi of the stomach exhibit signs characteristic of the situation which they occupy?* This question has an obvious reference to the *particular* DIAGNOISS of gastric scirrhus; but it is not, as we have intimated elsewhere, resolved by Dr. Chardel in a clear or satisfactory manner. The following brief outline of his observations on the subject, while it affords a few useful hints, will sufficiently illustrate the correctness of our opinion.

Vomiting and constipation do not, as some writers have erroneously stated, exclusively signalize pyloric scirrhus; they are common to all the varieties of the affection: nor can any positive inference be drawn from the site of the tumour. It is a very equivocal, and may even prove a delusive, sign; for scirrhus of the stomach frequently alters much the natural situation of the organ.* Scirrhus of the cardiac orifice seems to present some peculiar characters. These are, greater or less difficulty of swallowing solid and even liquid food, according to the degree of contraction of the orifice; a more speedy rejection of the food from the stomach (sometimes almost instantaneous) than in the other varieties of gastric scirrhus; a sort of vomiting of viscid saliva, probably resulting from the continual excitement of the œsophageal glands; and, lastly, the absence of an external tumour in the epigastric region, except the tumefaction extend from the cardia to the adjacent parts, when it may, perhaps, be obscurely felt, and less intolerance of pressure than in the central and pyloric varieties of the disease.

For the instruction of our younger readers, we shall here collect, and arrange under their respective heads, the leading diagnostic signs of the three varieties of gastric scirrhus. In doing this, we shall not only glean from the numerous facts exposed by Dr. Chardel, but avail ourselves of the little light with which our own ex-

---

* This fact is elucidated by the case of a woman, aged 74, who had a round tumour, four inches in diameter, and surrounded with several others of a smaller size, *below* the umbilicus. She was extremely emaciated; had a good appetite to the last; and was free from vomiting. On dissection, the stomach was found descending in an almost perpendicular direction to the pubis. The inferior third part of the organ, situated below the umbilicus, formed the tumour in question. There were several tuberculous glands towards both its curvatures, and on its anterior surface. The peritoneal coat, in the interstices between them, preserved its characteristic lustre. The pyloric orifice was contracted, almost disorganized; and the mucous membrane, in its vicinity, reduced to a blackish putrid pulp. The structure of the scirrhus was completely tuberculous. The liver, and the apex of the left ventricle of the heart, were converted into a fatty substance.

perience and observation on these diseases may serve to furnish us.

I. *Cardiac Scirrhus.—Leading symptoms.* Difficult deglutition; vomiting of the aliment immediately, or very soon, after its ingestion; abundant and viscid salivary discharge; emaciation constant, and probably more early and more strongly marked than in the succeeding varieties; an epigastric tumour rarely existing, or only obscurely felt; constipation more common than in the central variety; sometimes loss of voice. Its comparative infrequency with respect to pyloric scirrhus, and the influence which sex and age appear to exert in its production, should also be taken into the calculation. The site of the pain, and the effect of posture in inducing aggravation or relief, might serve, in combination with the foregoing signs, to fix the diagnosis upon a surer base. The latter observations will apply with equal correctness to the next variety.

II. *Central Scirrhus* may commonly admit of distinction, by the frequent absence of vomiting, which almost invariably signalizes the other two varieties; by the nearly constant appearance of an external tumour, generally about the centre of the epigastric region, or at least never so low down as is often seen in pyloric scirrhus; and by the infrequency of constipation with respect to the other varieties.

III. *Pyloric Scirrhus.—Leading symptoms.* Vomiting, usually from half an hour to two hours after the reception of food; an external tumour almost invariably perceptible, and sometimes so low down * as to suggest erroneous notions respecting its nature; constipation of the bowels rarely absent; occasionally dyspnœa. It occurs much more frequently, and commonly at a somewhat more advanced age, than the two preceding varieties; and its ravages are very generally confined to the male sex. From correct observation of the influence of posture upon the attendant pain, considerable light might, perhaps, be thrown on the diagnosis of pyloric scirrhus.

In the preceding sketch, we do not pretend to have fully and incontrovertibly fixed the diagnosis of the three varieties of gastric scirrhus. To do justice to this arduous

* The preceding note fully illustrates this important fact in the diagnosis of abdominal diseases. From not being duly apprized of it, physicians have been known to mistake scirrhus of the pylorus for an intestinal concretion.

subject, would demand the experience and scrutinizing eye of a Baillie, or a Corvisart. We have merely traced the outline, and marked the more prominent stages, of a route over this yet almost unexplored territory; which may be cleared, and rendered practicable, by future labourers. From the circumstance of cardiac and pyloric scirrhus frequently extending their ravages to the body of the stomach, it must always be very difficult, and often impossible, to distinguish either of these varieties from central scirrhus. But by any practitioner, possessing common powers of observation, exercising those powers upon a subject who is sufficiently intelligent to deliver a clear history of his symptoms and sensations, and availing himself of the feeble light which has just been elicited for his direction, correct discrimination of the cardiac and pyloric varieties of scirrhous stomach will, we are convinced, be, in almost every instance, attainable.

*Spasmodic vomiting.* We here pass from a consideration of the *particular,* to that of some points which interest the *general* DIAGNOSIS of gastric scirrhus. In order to propagate the knowledge of any disease, there is a necessity, not only that its leading characters should be precisely traced, but that other affections, which, from the resemblance exhibited by them, may be confounded with it, should be carefully pointed out and distinguished. This remark becomes especially momentous, when the disease in question is incurable; as, by mistaking for it others which are really susceptible of cure, we abuse the confidence of the patient, and exclude him from every prospect of the relief which he might otherwise obtain.

In this view, spasmodic vomiting, which is defined by Dr. Chardel to be an affection resulting from an "immediate lesion of the vital principle in the nerves of the stomach," demands peculiar consideration here; for no morbid condition so perfectly simulates gastric scirrhus as this. Eight cases are detailed, to shew the similarity of external character in these diseases, and to inculcate the propriety of reserve in forming our diagnosis of them; especially since both may arise from the operation of identical causes. We shall select, for the reader's instruction, that history which seems most strongly to illustrate the resemblance existing between scirrhus of the stomach and spasmodic vomiting.

A man, aged 50, of bilious temperament and strong constitution, had become subject, about his 36th year, to severe gastric pains during the process of digestion. They constituted a kind of paroxysm, which lasted twelve or fourteen days, and returned, somewhat regularly, every third or fourth month. From the employment of bitters and stomachics, transient relief sometimes ensued: yet the complaint periodically recurred; and, at other times, the man's health seemed excellent. Patience became his only remedy. The paroxysms continued long without aggravation; but, two years before, they excited violent efforts to vomit, which terminated in a copious rejection of mucus. In May, the year following, they returned with considerable vomiting, and thenceforth the health sensibly declined; digestion was obstructed; and nothing passed through the intestines. Hence the stomach became excessively distended; and, every five or six days, copious vomiting took place, which removed the acute pains felt by the patient in the stomach, right kidney, and dorsal portion of the spine. The calm continued till the stomach was again full. To these symptoms were added, eructations, frequently acid, and always of an unpleasant taste, and obstinate constipation. Purgatives and injections succeeded in procuring evacuations, of horrible fœtor, and afforded some relief, without, however, any suspension of the vomiting. The bowels soon resumed their usual torpidity; and, by the defect of nutrition, sleeplessness, and continual suffering, the patient was, in six months, reduced to a state of dreadful emaciation. He only sometimes quitted his bed for an arm chair; yet the mouth continued clean, and he had even an appetite when the pains were somewhat relieved. Purgatives, combined with bitters, opiates, the soap pill, and aperient drinks, were prescribed in vain. At the end of a year, the vomited matter was tinged with blackish streaks, which seemed to presage approaching death. Antispasmodics, baths, and suppositories, were then had recourse to; a blister was laid on the epigastric region, and renewed till the vomiting completely disappeared; and camphorated oil was rubbed in on the abdomen. Under this treatment, the cure was, in some months, completed, and convalescence established by generous diet and aromatic bitters.

From these cases, it appears, that all the symptoms of gastric scirrhus sometimes accompany spasmodic vomiting; and that the affection may even go so far as to produce the discharge of the blackish matter, which is supposed to announce ulceration of a scirrhus. Under such circumstances, the gastric glands are supposed to secrete a fluid analogous to that which colours the glands of the bronchiæ.

In proof of the fact that several other affections, widely different from scirrhus of the stomach, occasion also the vomiting of a blackish matter, two histories are quoted from the works of Morgagni and Portal.

An unusual induration of the abdominal muscles, particularly of the *recti*, when rendered strong by exertion; a tumour of the liver, an abscess in the posterior cavity of the omenta, enlargement of the pancreas, or a tubercular state of the mesenteric glands in the vicinity of the stomach, may readily be mistaken for scirrhus of that organ. Two of these positions, which the observer has most rarely an opportunity of verifying in practice, we shall illustrate by their correspondent examples.

A featherman, aged thirty-two, and much addicted to the pleasures of wine and women, was attacked, in February 1805, with deep-seated pain in the region of the stomach, followed by indigestion and frequent vomiting. These symptoms yielded not to a liberal use of antispasmodics. Four months after their appearance, a small tumour arose in the epigastrium, and progressively spread. Meanwhile, the violence of the symptoms was daily aggravated; and in December the patient entered *l'Hotel Dieu*. His emaciation was then extreme; and even the lightest aliment, soon after its ingestion, was rejected by vomiting. A hard substance, sinking under pressure, but speedily returning to its original state, was felt in the epigastrium. There were also copious diarrhœa, palpitation, quick and irregular pulse, paleness of the face, fluctuation in the abdomen, and œdema of the lower limbs. The disease was thought to be pyloric scirrhus in its most advanced stage, and aperients only were prescribed. The symptoms were exasperated on the morrow: sleeplessness; respiration frequent and laborious; pulse constantly irregular. Sedatives administered. On the ensuing day, respiration excessively disordered; pulse more irregular than ever, and intermittent; *facies hippocratica;* death.

On dissection, the brain, heart, and lungs were found healthy. The diaphragm, thrust upwards, encroached on the cavity of the thorax. The abdomen contained a quantity of serous fluid. The stomach, perfectly sound, was compressed and pushed anteriorly by an enormous abscess, formed in the posterior cavity of the omenta; the natural opening of which was completely obliterated. The portion of peritonæum constituting it was thickened, and had lost its wonted transparency; but this membrane was otherwise healthy throughout. The abscess contained a white, thick, grumous pus, perfectly resembling that of cellular structure, and in quantity about three English pints. The intestines were contracted.

An embroiderer, aged thirty-eight, was admitted into the Cochin Hospital in November 1807. All which could be learned respecting her was, that, for six months previously, she had been tormented by gastric pains, unattended with vomiting; and that, during the last fortnight, they had given place to copious diarrhœa. The state of the abdominal viscera was, at first, made out but obscurely by examination: for it induced violent contraction of the muscular parietes. This irritability, however, was shortly removed by fomentations; and several tumours, directed transversely towards the stomach, and appearing to terminate at the left iliac fossa, were discovered. One of these tumours, considerably larger than the rest, was situated a little to the right, as much in the umbilical as in the epigastric region. The emaciation of the woman, and expression of her countenance, announced impending death; which soon afterwards took place.

On dissection, a hard, white tumour, of homogeneous structure, and as large as the fist, was found developed in the transverse mesocolon. It emitted, on pressure, a sort of solid cheesy matter; and was evidently nothing more than a tuberculous gland. The compression which it exercised on the stomach, near the pylorus, had produced a somewhat firm vegetation of the mucous membrane, extending towards the lesser curvature. The pylorus itself was slightly contracted. The peritoneal coat preserved its peculiar lustre. A great number of tumours, resembling the first, but smaller, was seen on the mesentery, and in the substance of the liver. The latter organ extended into the left hypochondrium, and depressed the stomach: its parenchyma was apparently sound. The whole capillary system of the abdomen was injected, but nothing unusual detected in the cranium or thorax.

A simple nervous affection may sometimes impose itself upon the physician for a gastric tumour. In a delicate boy, of thirteen years, who had been attacked with very severe pains in the head and stomach, consequent on the abuse of acids, the epigastric and hypochondriac regions exhibited a considerable swelling. Pressure on these parts was almost intolerable. Sedatives afforded no relief. The lad was eventually cured by a liberal employment of musk.

Scirrhi of the stomach sometimes ulcerate without having induced severe suffering. An instance of this occurs in the work of Morgagni (*Epist.* xxx. *Observ.* 2). Hence, the mild character of the epigastric pain affords but a feeble inference in favour of spasmodic vomiting. On the other hand, such violent pain occasionally accompanies this kind of vomiting, that the epigastric region is incapable of sustaining the slightest touch with impunity. This excessive sensibility, indeed, seems rather to characterize nervous affections than gastric scirrhus.

Although no symptom decidedly signalizes spasmodic vomiting; yet, from an assemblage of signs, may frequently be formed an opinion approaching to certainty. Thus, if a young person* be attacked with vomiting suddenly, consequent on a severe nervous commotion, recurring periodically, and long protracted without deterioration of the system, or the developement of a tumour in the epigastric region, it may be considered as not resulting from organic lesion. Spasmodic vomiting probably often terminates in the production of gastric scirrhus, which early attention might have served to obviate.

*Vomiting, dependent on various causes.* Other kinds of vomiting, besides the spasmodic, may be confounded with that which results from scirrhous stomach. The relations existing between this organ and the uterus, frequently induce obstinate vomiting in females who menstruate irregularly: but other symptoms, completely foreign to gastric lesion, are then usually developed, and prevent error. In general, all vomiting consequent on deranged menstruation, especially if it continue long without producing extreme marasmus, may be looked upon as totally unconnected with scirrhus of the stomach. Obstinate vomiting, such as to delude a superficial observer, may also result from an affection of the urinary passages; from irritation of any kind, receding from the circumference to the stomach; from displacement (hernia) of the gastric organ; or from chronic inflammation of its mucous membrane. All the cases, respectively illustrative of these points, we have not time, nor, indeed, would it be worth our while to introduce. We therefore prefer the last, as

* Scirrhus of the stomach, as all the cases accumulated by Dr. Chardel incontestibly prove, rarely occurs till between the thirtieth and fortieth year. Hence, youth itself constitutes a strong presumption against the existence of the disease.

affording a good specimen of a disease, by no means infrequent in occurrence; and which it has been our lot, more than once, to see mistaken for some morbid affection of the liver or pancreas, and confirmed, or even rendered speedily fatal, by the practice founded on an erroneous diagnosis.

A cook, aged sixty-one, long addicted to potations of the strongest brandy, was, at last, reduced to extreme weakness. His whole body became remarkably pale, with a tendency to puffiness. Diarrhœa, after tormenting him for six months, spontaneously ceased, and was succeeded by vomiting, which no remedy would control. It had lasted three months; when, in August 1807, the man was admitted into the Cochin Hospital. He then exhibited an advanced state of cachexia, and was plunged into a sort of moral and physical insensibility, truly remarkable. The abdomen was destitute throughout of tumour or induration, and bore pressure with impunity. The tongue was clean, but of a bright red colour, and, as it were, inflamed. The thirst was not intense: the bowels were constipated. Medicine of every kind was obstinately rejected by the patient, in the fear that it would excite vomiting. He invariably said that he felt well. Wine, brandy, broth, and soup, composed his diet. The inflammatory condition of the tongue, and absence of epigastric tumour, seemed to indicate that the vomiting depended on inflammation of the mucous membrane of the stomach. In this state of apathy, the man lingered till the beginning of October. Towards the close of life, a kind of tumour was distinguished in the pyloric region, where pain was excited by pressure: and obscurity thus involved the nature of the case. On dissection, the abdomen contained a little reddish serum. The stomach, otherwise sound, displayed traces of inflammation, most decided about the cardia and pylorus, and exclusively confined to the mucous membrane; the liver was large.* A prodigious quantity of fat loaded the omenta and mesentery; and was accumulated round the heart and kidnies. The intestines, although healthy, were contracted so as to appear very small. The arch of the colon contained some indurated fæces, which had been mistaken for a tumour of the pylorus: the other viscera exhibited nothing un-

* The word is *gras, fat,* in the original. It is probably a mis-print for *gros.* Rev.

usual. All the muscles were remarkably pale, and discoloured.

*General Remarks (Généralités) on the* TREATMENT *of scirrhous Affections of the Stomach.* We shall close this long Review, from which we would fain hope that the British practitioner will derive somewhat of both interest and information, with a nearly literal transcript of Dr. Chardel's concluding section.

Gastric scirrhus can only be successfully combated by prevention. Once developed, it is beyond the resources of art. But, most commonly, it is announced by symptoms so equivocal, that its character may be readily mistaken. Cases may, at first, exhibit, according to their causes, different symptoms and particular indications; yet they all terminate in resembling each other, and in requiring the adoption of palliative remedies. If, for example, an inflammatory state of the stomach exist, the employment of the antiphlogistic plan is indicated, in order to obviate congestion of the membranes, inflammation of the lymphatics, and the consequent production of tubercles. But, afterwards, the powerful aid of derivation may be advantageously had recourse to, without, however, forgetting the dangers of a too violent reaction. Might not mercury also, which has been recommended in chronic thickening of the parietes of the bladder, constitute an useful remedy in these cases? Blistering of the epigastric region might be usefully employed against a non-inflammatory irritation fixed on the stomach. It becomes necessary to operate both locally and generally on the system, when the disease depends upon a peculiar modification of the lymphatics.

Scirrhus, once formed, forbids the adoption of a curative plan. The efforts of art should then merely be to palliate symptoms, and to retard, as much as possible, the ulceration of the tumour. Opium and anti-spasmodics are the only efficacious remedies which medicine here offers; they serve to mitigate the pain, and even sometimes to suspend the vomiting, by diminishing the irritability of the stomach.

Gentle aperients and injections frequently render the constipation less obstinate, and are thus productive of transient relief. Topical emollients become likewise very useful, if severe pain be felt; for it is important to arrest the progress of inflammation, by which the tumour would be speedily disorganized.

Œdema and ascites are frequent consequences of the state of exhaustion to which the patient is reduced from defective nutrition. Aperients are obviously here of little use; their exhibition may even prove dangerous, by unduly irritating the stomach; and hence it demands much precaution. The diarrhœa, which often supervenes in the concluding stage of the disease, will be best controlled by mucilaginous drinks and opiates; but all that medicine can then accomplish, is the prolongation of a wretched existence.

With respect to diet, substances the most nutritive, digestible, and unirritating, should, in general, be preferred. Nevertheless, the caprices of appetite may, to a certain point, be very properly indulged.

Long details on the treatment of scirrhous affections of the stomach come not within the pale of a simple monograph: and, in the present state of our knowledge, general precepts can alone be delivered. The application of these to individual cases obviously devolves on the physician himself.

